# Regulation of type 1 diabetes development and B-cell activation in nonobese diabetic mice by early life exposure to a diabetogenic environment

**DOI:** 10.1371/journal.pone.0181964

**Published:** 2017-08-03

**Authors:** Alessandra De Riva, Maja Wållberg, Francesca Ronchi, Richard Coulson, Andrew Sage, Lucy Thorne, Ian Goodfellow, Kathy D. McCoy, Miyuki Azuma, Anne Cooke, Robert Busch

**Affiliations:** 1 Department of Medicine, University of Cambridge, Cambridge, United Kingdom; 2 Department of Pathology, University of Cambridge, Cambridge, United Kingdom; 3 Maurice Müller Laboratories (DKF), Universitätsklinik für Viszerale Chirurgie und Medizin Inselspital, University of Bern, Bern, Switzerland; 4 Department of Molecular Immunology, Graduate School, Tokyo Medical and Dental University, Tokyo, Japan; 5 Department of Life Sciences, University of Roehampton, London, United Kingdom; Children's Hospital Boston, UNITED STATES

## Abstract

Microbes, including viruses, influence type 1 diabetes (T1D) development, but many such influences remain undefined. Previous work on underlying immune mechanisms has focussed on cytokines and T cells. Here, we compared two nonobese diabetic (NOD) mouse colonies, NOD^low^ and NOD^high^, differing markedly in their cumulative T1D incidence (22% vs. 90% by 30 weeks in females). NOD^high^ mice harbored more complex intestinal microbiota, including several pathobionts; both colonies harbored segmented filamentous bacteria (SFB), thought to suppress T1D. Young NOD^high^ females had increased B-cell activation in their mesenteric lymph nodes. These phenotypes were transmissible. Co-housing of NOD^low^ with NOD^high^ mice after weaning did not change T1D development, but T1D incidence was increased in female offspring of co-housed NOD^low^ mice, which were exposed to the NOD^high^ environment both before and after weaning. These offspring also acquired microbiota and B-cell activation approaching those of NOD^high^ mice. In NOD^low^ females, the low rate of T1D was unaffected by cyclophosphamide but increased by PD-L1 blockade. Thus, environmental exposures that are innocuous later in life may promote T1D progression if acquired early during immune development, possibly by altering B-cell activation and/or PD-L1 function. Moreover, T1D suppression in NOD mice by SFB may depend on the presence of other microbial influences. The complexity of microbial immune regulation revealed in this murine model may also be relevant to the environmental regulation of human T1D.

## Introduction

Type 1 Diabetes (T1D) is a chronic autoimmune disease in which pancreatic beta-cells are destroyed by self-reactive lymphocytes, resulting in insulin deficiency and hyperglycaemia. T1D development in genetically susceptible individuals [1] depends on environmental factors, consistent with the modest concordance for T1D in monozygotic twins (50–60%)[2]. Importantly, the incidence of T1D has been rising at 3–4% per year in European children in the last 15 years [3, 4], and this cannot be explained on the basis of genetic changes in the population.

Improved sanitation and hygiene, alongside rising pollution, are thought to have altered immune regulation by the environment in industrialized countries, both in the context of allergic [5] and autoimmune disease [6] (“Hygiene Hypothesis”). Regulation of autoimmunity by infection was also demonstrated by early work showing that malaria-infected (NZB × NZW) F1 mice were protected from lupus nephritis [7]. Consistent with microbial regulation of autoimmunity, recent studies have reported differences in the intestinal microbiota between patients with new-onset T1D, autoantibody-positive individuals at risk, first-degree relatives, and healthy controls [8–13], although the identification of causal influences remains in its infancy.

Environmental factors, and specifically the intestinal microbiota, also are critical in the nonobese diabetic (NOD) mouse, a well-characterized model of T1D [14], which shares many genetic risk determinants with human T1D. Consistent with the Hygiene Hypothesis, germ-free NOD mice develop T1D with a high incidence in both males and females [15–17], whereas T1D development is more variable, sex-dependent, and often lower in conventional facilities [18, 19]. The Hygiene Hypothesis is also consistent with experiments showing that intestinal metazoan parasites [20], bacterial pathogens [21], and commensal segmented filamentous bacteria (SFB) [22] suppress the development of T1D, often by regulating the cytokine milieu for T-cell activation and regulation [23]. In contrast, several viral infections promote T1D development; given that viral infections stimulate Th1 immunity, this is consistent with the notion that autoimmune destruction of pancreatic islets is a Th1-dependent process [23]. Other factors that influence T1D development in NOD mice, such as sex [24], drinking water acidity [25], and antibiotic treatment [26, 27], also appear to act *via* the gut microbiota. Thus, T1D incidence rates in this model are thought to depend on a balance of T1D-promoting and -inhibiting microbial influences on T-cell immunity. Given that microbiota vary widely between animal facilities worldwide, it is not surprising that T1D incidence rates also vary widely between such facilities [19]. In many cases the relevant microbial and viral factors and underlying immune mechanisms remain undefined.

We previously reported on two colonies of NOD mice, which had been derived from shared founders but maintained separately for many years [28]. Whereas only ≈ 20% of females from one colony, called “NOD^low^”, developed T1D by 30 weeks of age, ≈ 90% of females in the other colony, called “NOD^high^”, became diabetic. Here, we investigated environmental and immunophenotypic differences between the two colonies, as well as the transmissibility of the colony differences in T1D development. Together, these studies revealed further complexities in the environmental regulation of T1D and novel features of the underlying immune mechanisms.

## Materials and methods

### Mice

Animal studies were performed according to institutional and national guidelines under UK Home Office Project Licenses 80/2156 and 80/2442. The two NOD sister colonies used in this study were kindly provided by Prof Linda Wicker and bred under specific pathogen–free (SPF) conditions either at the Centre for Biomedical Services, University of Cambridge, named “NOD^low^”, or at Biological Services Unit of the Department of Pathology, University of Cambridge, named “NOD^high^”. The NOD^low^ colony has consistently maintained a T1D incidence of ≈ 22% (measured in females up to 30 weeks of age). The NOD^high^ colony was derived from NOD^low^ founders around 2002; its T1D incidence rose gradually after the founders were moved to Biological Services Unit of the Department of Pathology, and has subsequently remained high for several years (≥ 90% by 30 weeks of age in females). NOD genotype was authenticated in a breeding trio from each colony using an array of 384 SNPs, spaced ≈ 7 MB apart across the entire genome, which distinguish common inbred mouse strains (Charles River Laboratories). Results from one of the SNPs in the NOD^low^ analysis were technically uninformative; all other results were consistent with homozygous NOD genotype. For incidence studies, animals were aged to 30 weeks and observed daily for signs of diabetes, such as polyuria, and of other signs of ill health; diabetes was confirmed by measurement of glucosuria, using Diastix (Bayer Diagnostics). Glycosuria is a physiologically relevant endpoint, indicating that blood glucose levels have exceeded the capacity for renal reabsorption. This is thought to occur at ca. 300 mg/dl plasma glucose, i.e., at twice the normal levels [29]. As a humane endpoint, development of diabetes triggered prompt euthanasia by CO_2_ anesthesia followed by cervical dislocation. There were no unexplained deaths or other signs of ill health.

Animals were maintained in individually ventilated cages and received water *ad libitum*. Both colonies were maintained by breeding the first litter in each generation and initially kept on the same diet (RM3 [E] irradiated diet, Special Diet Services, Witham, UK); breeders received the same diet supplemented with 7% oil. In 2012, the supplier for the diet used in the NOD^low^ colony was changed (no. 105 diet, SAFE SAS, Augy, France) without a change in incidence. Routine veterinary microbiological screening of sentinel mice in both colonies was carried out according to current recommendations [30, 31] of the Federation of European Laboratory Animal Sciences Associations, or better, as discussed in the text (B&K Diagnostics).

### Insulitis scores

Pancreata were harvested, preserved in 4% v/v in paraformaldehyde solution, and processed for wax histology. Five-micrometer sections were taken at three levels and stained with hematoxylin and eosin. Total islets per section were counted and the degree of cellular infiltration was scored as follows: no infiltration; peri-insulitis = infiltrate present as a single cluster of immune cells at one pole of the islet; intra-insulitis = diffuse pattern of infiltration within the islet.

### PD-L1 inhibition

As described previously [32], mice were injected intra-peritoneally with a PD-L1 blocking monoclonal antibody (clone MIH5) at a dose of 2 mg/mouse. Development of diabetes was followed for 24 days post injection.

### Cyclophosphamide treatment

As described previously [33], cyclophosphamide (Endoxana, Baxter Healthcare) was prepared in 0.9% normal saline at 20 mg/ml immediately before intra-peritoneal administration. Development of diabetes was followed for 24 days post injection.

### Cell isolation, antibodies and flow cytometry

Lymphocyte cell suspensions were prepared in complete medium RPMI (Lonza) by lymph node disruption using 100 μm nylon mesh (BD Falcon).

For flow cytometry, the following antibodies were purchased from Biolegend: anti CD3-PE-Cy7 (clone 145-2C11; anti CD4-PerCP (clone RM4-5); anti CD69-FITC (clone H1.2F3). Anti B220-eFluor450 (clone RA3-6B2) was from eBioscience. For anti Foxp3-PE (clone FJK-16s) staining, cells were first fixed and permeabilized using the Foxp3 / Transcription Factor Staining Buffer Set from eBioscience following the manufacturer’s instructions. Analytical flow cytometry was conducted using a FACSCanto II (BD Biosciences), and the data were processed using FlowJo software (Tree Star).

### Anti-BAFF ELISA

Serum BAFF was measured by standard colorimetric ELISA using rat anti-mouse BAFF (clone 121808) as capture antibody and biotinylated goat-anti mouse BAFF as detection antibody (both R&D Systems). BAFF concentration was determined using a standard curve of recombinant mouse BAFF (R&D Systems).

### Bacterial 16S DNA detection in feces

Fecal samples were collected from mice and stored at -80°C until processing. DNA was isolated using a DNA Qiagen kit following manufacturer’s instructions. Bacterial DNA was detected by PCR analysis for 16S ribosomal RNA genes, using the following primers: for Existing Universal Bacterial (EUB), forward primer CGGCAACGAGCGCAACCC and reverse primer CCATTGTAGCACGTGTGTAGCC were used as described previously [34]. For *Helicobacter hepaticus*, forward primer ATGGGTAAGAAAATAGCAAAAAGATTGCAA and reverse primer CTATTTCATATCCATAAGCTCTTGAGAATC were used as described previously [35]. For SFB, forward primer GACGCTGAGGCATGAGAGCAT and reverse primer GACGGCACGGATTGTTATTCA were used as described previously [22]. Quantitative PCR was carried out using KAPA SYBER FAST qPCR Kit (KAPABIOSYSTEMS) and following the manufacturer’s instructions on the ABI PRISM 7000 Sequence detection system (Applied Biosystems). PCR signals for species-specific 16S rRNA genes were normalized to EUB. For qualitative analysis, the 705 bp PCR products were run on a 2% agarose (BIOLINE) gel in TEA; the band size was determined against the Gene Ruler 100 bp DNA Ladder (Thermo Scientific).

### Co-housing experiment

Three-week-old NOD^low^ mice were transferred from the Centre for Biomedical Services, University of Cambridge to the Biological Services Unit of the Department of Pathology, University of Cambridge. After resting for 48 hours, mice were co-housed with age- and sex-matched NOD^high^ mice and scored for T1D up to 30 weeks of age. At 6 weeks of age, some of the co-housed animals of NOD^low^ and NOD^high^ origin were separated from each other and set up for breeding. Female offspring from both colonies of origin were then co-housed once more at weaning age (three weeks) and scored for T1D until 30 weeks of age.

### Fecal matter transfer experiments

Three-week-old NOD^low^ females were gavaged with diluted fecal contents from NOD^high^ female donors. Briefly, fecal matter was collected from three12-week-old NOD^high^ females and resuspended in 50 volumes of sterile water. After filtering the suspension with a 70 μm nylon strainer, 250 μl of this suspension was given to each recipient by oral gavage using a 24G round tip gavage needle. Recipients received another dose of fecal matter after resting for 48 hours. Fresh fecal samples from individual recipients were collected at various times, starting from two weeks post transfer. Mice were maintained in an isolator dedicated to this study and scored for T1D up to 30 weeks of age.

### IonTorrent sequencing and analysis

DNA from fecal samples was extracted using QIAamp FAST DNA Stool Kit (QIAGEN) with additional digestion in the presence of lysozyme (20 mg/ml). Variable V5 and V6 regions of the 16S rRNA gene were amplified from DNA from fecal samples using the barcoded forward fusion primer *CCATCTCATCCCTGCGTGTCTCCGACTCAG*ATTAGATACCCYGGTAGTCC in combination with the reverse fusion primer *CCTCTCTATGGGCAGTCGGTGAT*ACGAGCTGACGACARCCATG. The sequences include IonTorrent PGM-specific adaptors (in italics) that are required for high throughput sequencing. The PCR-amplified 16S V5-V6 amplicons were purified from agarose gels using Qiagen Gel extraction kit (according to manufacturer’s instructions) and then prepared for sequencing on the IonTorrent PGM system using Ion PGM^TM^ Template OT2 400 Kit and Ion PGM^TM^ Sequencing 400 Kit according to the manufacturer’s instructions (Life Technologies). The number of reads obtained per sample was between 7000 and 65000. Data analysis was performed using the QIIME pipeline version 1.8.0. Operational taxonomic units (OTU) were picked using uclust [36] and the latest greengenes database (http://greengenes.secondgenome.com).

### Statistical analysis

Statistical analyses were performed using GraphPad Prism. Kaplan–Meier analysis of cumulative disease incidence was performed, and incidence curves were compared by the log-rank test. Differences in immuno-phenotypes were evaluated by unpaired, two-tailed Student *t* test, or one- or two-way-ANOVA, as appropriate.

Statistical analysis of metagenomic data using R was performed using the QIIME pipeline [37]. Briefly, the 'phyloseq' and 'DESeq2' packages were used. The function 'DESeq' performs the differential taxon-count analysis based on the negative binomial distribution, and uses the OTU table as its input. It returns the estimated log_2_-fold-changes and p values (Wald test) corrected for multiple testing for the comparison of interest (Benjamini & Hochberg correction). Principal coordinates analysis, a standard biostatistics technique [38, 39], was performed in order to represent the dissimilarity between any two microbiomes (measured by their unweighted UniFrac distances) in two dimensions.

## Results

### Natural history and genetics of NOD^low^ and NOD^high^ colonies

**Fig 1:**
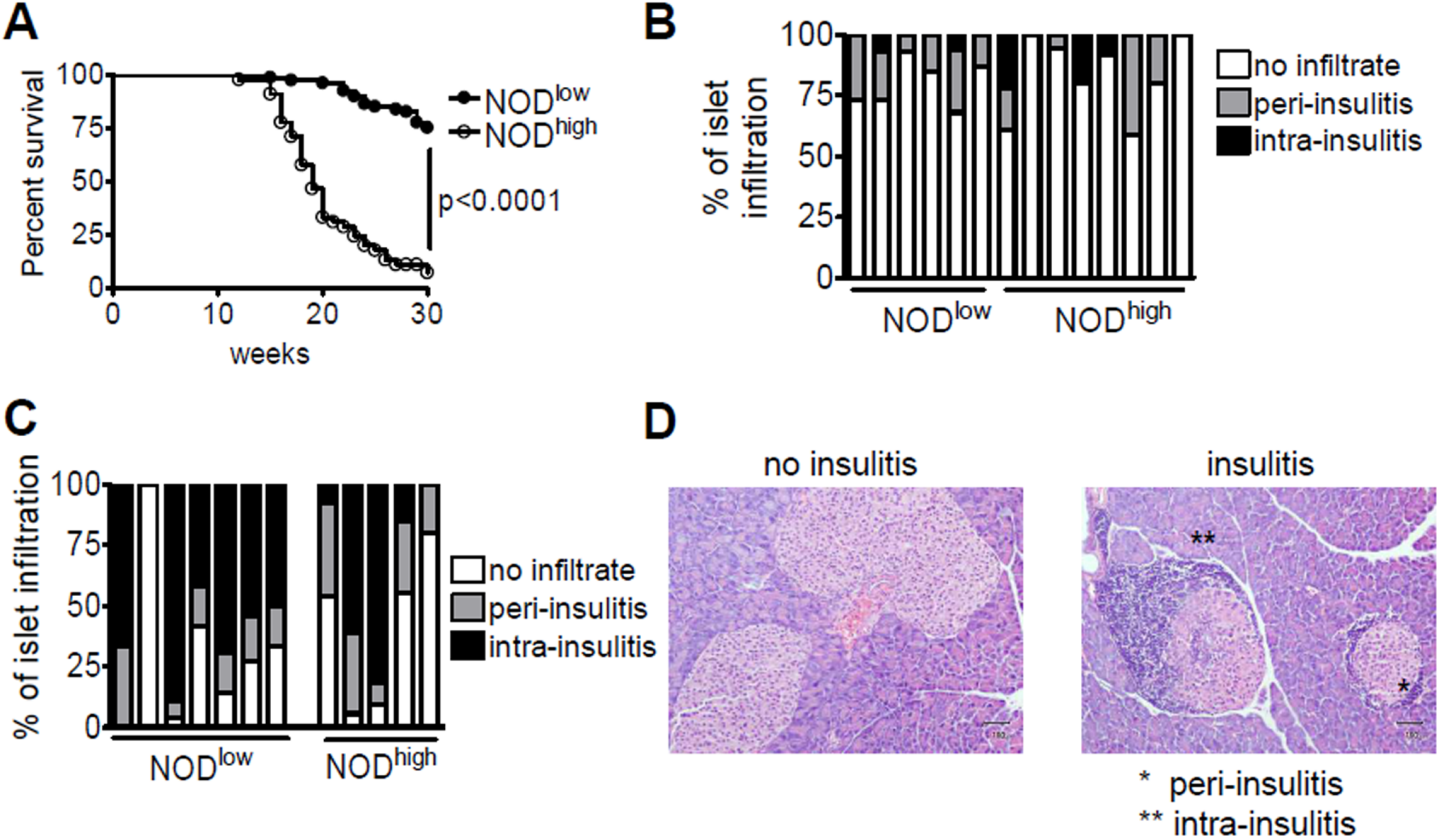
Characterisation of NOD^low^ and NOD^high^ colonies. (**A**) Kaplan-Meier survival curves showing diabetes-free survival up 30 weeks of age in 85 NOD^low^ (black circles) and 45 NOD^high^ (white circles) female mice. Incidence curves were compared by the log-rank test. From [28] with permission; Copyright 2013, The American Association of Immunologists, Inc. (**B**) and (**C**) Percent of islets exhibiting no insulitis, peri- or intra-insulitis in six NOD^low^ vs. eight NOD^high^ 6-week-old female mice (**B**), or in seven NOD^low^ vs. five NOD^high^ 30-week-old female mice (**C**). Each bar represents analysis of three pancreatic sections from an individual mouse. (**D**) Examples of H&E stained pancreatic sections from 30-week-old NOD^low^ females showing, on the left, two islets without insulitis and, on the right, one islet each with peri-insulitis (*) and intra-insulitis (**).

We have previously reported that two sister NOD colonies kept in different animal houses develop T1D with low (NOD^low^) and high (NOD^high^) cumulative incidence in females [28] (shown here again in Fig 1A). The difference spans the range observed in NOD colonies worldwide [19]. In both colonies, male incidence was lower, consistent with findings in many SPF or conventionally-housed NOD colonies [19, 40, 41]. Accordingly, subsequent microbiological, phenotypic and disease incidence studies were carried out on females only. NOD^low^ mice had been housed in an SPF facility with individually ventilated cages and maintained stable low rates of disease incidence for at least a decade. The NOD^high^ colony was derived from NOD^low^ founders around 2002, when founders were transferred to a different facility with open cages; incidence rose gradually over the next two years and subsequently remained stable at a high level.

In order to exclude the possibility of genetic contamination with other common inbred strains, genotyping was performed in a breeding trio from each colony by screening 364 SNPs spread across the entire genome, spaced ≈ 7 MB apart on average, and representing common inbred mouse strains. All informative SNPs were consistent with homozygosity for the NOD genotype.

To assess whether the difference in development of T1D in the two colonies reflected different degrees of insulitis, we examined H&E-stained pancreatic sections. The level of infiltration in the pancreatic islets was similar between NOD^high^ and NOD^low^ mice, both at 6 weeks (Fig 1B) and at 30 weeks of age (Fig 1C and 1D), but overall there was more insulitis in the aged mice. In summary, the difference in diabetes development was not due to genetic contamination, nor was it related to differences in the degree of insulitis between the two colonies.

### Low T1D incidence in NOD^low^ mice is unaffected by cyclophosphamide treatment but depends on PD-L1

**Fig 2:**
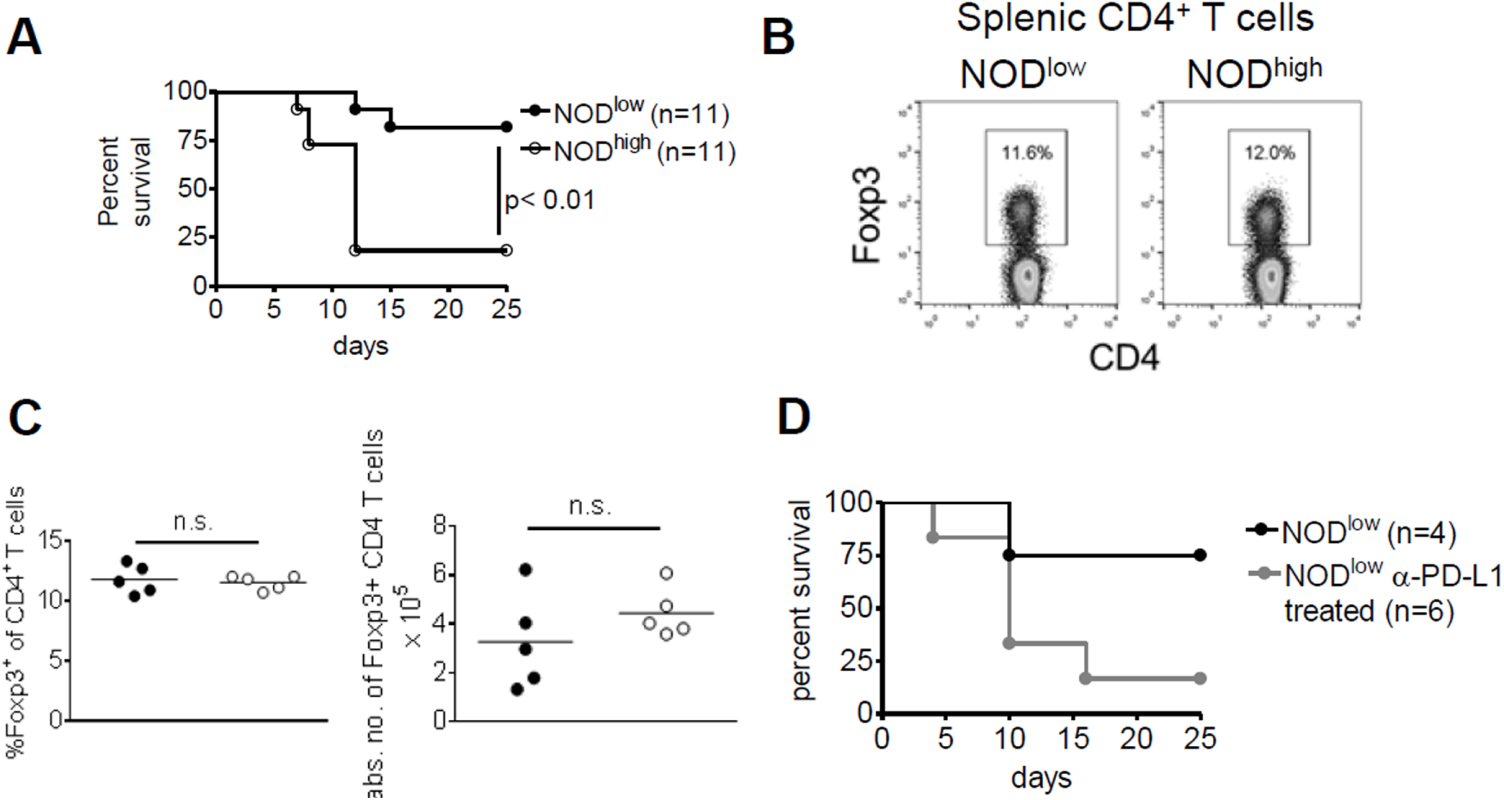
T1D is induced in NOD^low^ mice by anti-PD-L1 antibody, but not by cyclophosphamide. (**A**) Normoglycaemic (pre-diabetic) NOD^low^ (black circles) and NOD^high^ (white circles) female mice were treated at 16–18 weeks of age with cyclophosphamide, and T1D development was monitored daily up to 25 days post intra-peritoneal injection. (**B**) Representative FACS plots for CD4 (x-axis) vs. FoxP3 (y-axis) on gated CD4^+^ T cells from NOD^low^ (left) and NOD^high^ (right) spleens. (**C**) Percentages (left) and absolute numbers (right) of FoxP3^+^ CD4 T cells from pancreatic lymph nodes of NOD^low^ (full circles) and NOD^high^ (white circles) mice. Statistical analysis was performed using Student’s t test. (**D**) Normoglycaemic (pre-diabetic) NOD^low^ females were treated with anti- (α-) PD-L1 (clone MIH5, grey circles) or PBS (black circles) at 12–14 weeks of age, and T1D development was monitored daily up to 25 days post intra-peritoneal injection. In (A) and (D), diabetes-free survival was compared between groups by Kaplan-Meier analysis and log rank test.

Next, we examined mechanisms that could prevent progression to T1D in NOD^low^ mice. In some settings, cyclophosphamide has been shown to promote the rapid progression to T1D in pre-diabetic NOD mice by depleting regulatory T cells [33, 42]. We confirmed our previous findings [33] that most cyclophosphamide-treated pre-diabetic NOD^high^ females rapidly progressed to overt T1D within two weeks (Fig 2A). This contrasted with the variable age of onset of T1D in untreated NOD^high^ females, between 12 and 30 weeks of age (cf. Fig 1A). Surprisingly, however, the same treatment failed to trigger T1D in pre-diabetic NOD^low^ females (Fig 2A). Moreover, by flow cytometry we established that percentages and absolute numbers of CD4^+^ Foxp3^+^ T cells were similar in NOD^low^ and NOD^high^ mice (Fig 2B and 2C). Together, these findings suggest that the low rate of T1D development in NOD^low^ mice does not depend on CD4^+^ T_reg_ cells.

Anti-PD-L1 antibody treatment affords another means of overcoming resistance to autoimmunity [43, 44] by disrupting inhibitory signals that prevent activation and effector functions of pathogenic T cells [45, 46]. Blockade of this pathway by intra-peritoneal injection of anti-PD-L1 antibody rapidly precipitated T1D in old pre-diabetic NOD^low^ mice (Fig 2D). The comparison to a small control group (n = 4) in Fig 2D did not reach statistical significance (p = 0.09, log rank test), but the low incidence of T1D in the control group was in line with incidence in the NOD^low^ colony (see Fig 1A), whereas the rapid rise in T1D incidence (to 5/6 animals = 83%) within 3 weeks of anti-PD-L1 treatment was clearly different. We concluded that in NOD^low^ mice autoimmunity is maintained in a latent, sub-clinical state by PD-1/PD-L1 signalling.

### Environmental differences between NOD^high^ and NOD^low^ colonies

Next, we examined environmental differences that might explain the colony difference in T1D development. Both colonies were maintained on the same 12h/12h light/dark cycle, and kept on the same diet initially; the diet was modified in the NOD^low^ colony in 2012 without incidence being affected. The drinking water in both animal facilities was neutral.

**Fig 3:**
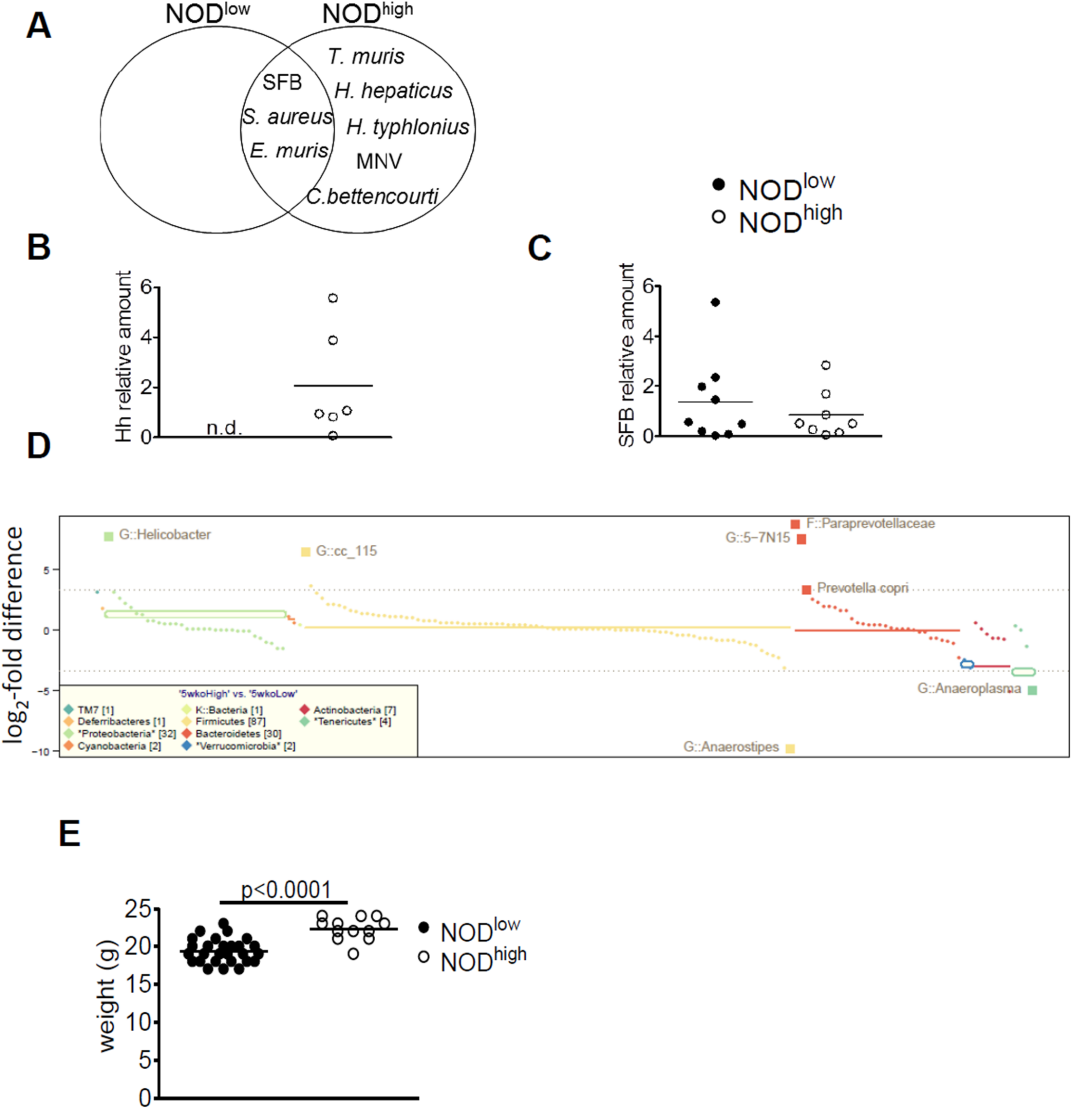
Microbiological characterisation of female NOD^low^ and NOD^high^ mice. (**A**) Venn diagram depicting microorganisms consistently detected by routine health screens in sentinel mice from the NOD^low^ and NOD^high^ colonies (also includes the results of screening for SFB). None of the other viruses, bacteria or parasites that are routinely tested under FELASA guidelines [30, 31] were detected in either colony. (**B** and **C**) Quantification by qPCR (normalized to EUB) of *H*. *hepaticus* (**B**) and SFB (**C**) in the feces of individual NOD^low^ (black circles) and NOD^high^ (white circles) mice; horizontal bars represent means. Species-specific primers for the 16S RNA gene were used. (**D**) Metagenomic analysis of bacterial 16S rRNA genes from NOD^low^ and NOD^high^ females at 5 weeks of age. Different bacterial clades are color-coded, and log_2_-fold mean differences between the detection frequencies in NOD^high^ and NOD^low^ mice shown on the y-axis; positive values show over-representation in the NOD^high^ colony. The dotted horizontal lines represent tenfold differences in either direction; significant colony differences (p < 0.05 after correcting for multiple comparisons) of tenfold or greater are shown as large squares with their names given. (**E**) Weights of individual age-matched NOD^low^ and NOD^high^ females (symbols as in **B/C**); means (horizontal lines) were compared by Student’s t test.

Given the importance of microbes in regulating autoimmunity, we reviewed veterinary screening reports of the two colonies. Differences in microbial colonisation and persistent viral infection were apparent (summarized in Fig 3A). *Helicobacter (H*.*) hepaticus* and *H*. *typhlonius* were detected in NOD^high^, but not in NOD^low^ mice. The difference in *H*. *hepaticus* infection was confirmed by PCR analysis of feces from the two colonies: 5/6 NOD^high^ animals tested positive for *H*. *hepaticus*-specific 16S RNA genes, but none of the NOD^low^ animals did (Fig 3B).

Veterinary screening for murine norovirus (MNV), in accordance with the 2014 revised FELASA guidelines [31], was positive in the NOD^high^ colony, as well (see Supporting Information, S1 Appendix). MNV was known to have been introduced into this colony years after the animals had already acquired their high cumulative T1D incidence. Therefore, MNV could not have been the cause of the rise in incidence. We nonetheless addressed the formal possibility that MNV might be diabetogenic, but observed no rise in T1D after infecting NOD^low^ mice with MNV strain 3 (see Supporting Information, S1 Appendix). Thus, neither the natural history of the NOD^high^ colony nor the outcomes of deliberate infection suggest any diabetogenic role for MNV in this system.

Even though they are not included in routine veterinary screening, we used PCR to test for segmented filamentous bacteria (SFB), whose presence in NOD intestines reportedly correlates with protection from T1D [22]. However, feces from both colonies harbored similar levels of SFB, as judged by quantitative PCR analysis (Fig 3C). This finding ruled out differences in SFB colonisation as the explanation for the difference in diabetes incidence.

A more comprehensive survey of microbial differences between the two NOD colonies was then performed. High-throughput sequencing of 16S rRNA genes confirmed that NOD^high^ but not NOD^low^ mice were colonized by *Helicobacter* spp. (Fig 3D and S1 Fig). This analysis also detected differences in the abundance of several other bacterial taxa, some of which appeared highly significant (Paraprevotellaceae, *Anaerostipes*), whereas others were more modest (*Prevotella copri*). Most represented gains by the NOD^high^ colony, but there were a few losses, as well (Fig 3D). The overall abundance of major clades barely differed between the colonies (horizontal lines in Fig 3D).

*Helicobacter* spp. and MNV have been associated with intestinal inflammation in other model systems (cf. Discussion), but in the NOD^high^ colony, we found no inflammation of the intestinal lining upon histological examination. Consistent with this, young animals in this colony exhibited no failure to thrive; indeed, despite being on the same diet, they gained slightly more weight than age-matched NOD^low^ animals (Fig 3E).

In summary, the microbiota of NOD^low^ mice differed markedly from those of the NOD^high^ colony. Compared to NOD^low^ mice, NOD^high^ mice had lost a few species while gaining several others, including the known pathobionts, MNV and two *Helicobacter* species. The presence of SFB in the NOD^high^ colony raised the possibility that any suppressive influence of SFB on autoimmunity was overcome by other, diabetes-promoting factors in its environment.

### Increased cellularity and B-cell activation in mesenteric lymph nodes from NOD^high^ mice

**Fig 4:**
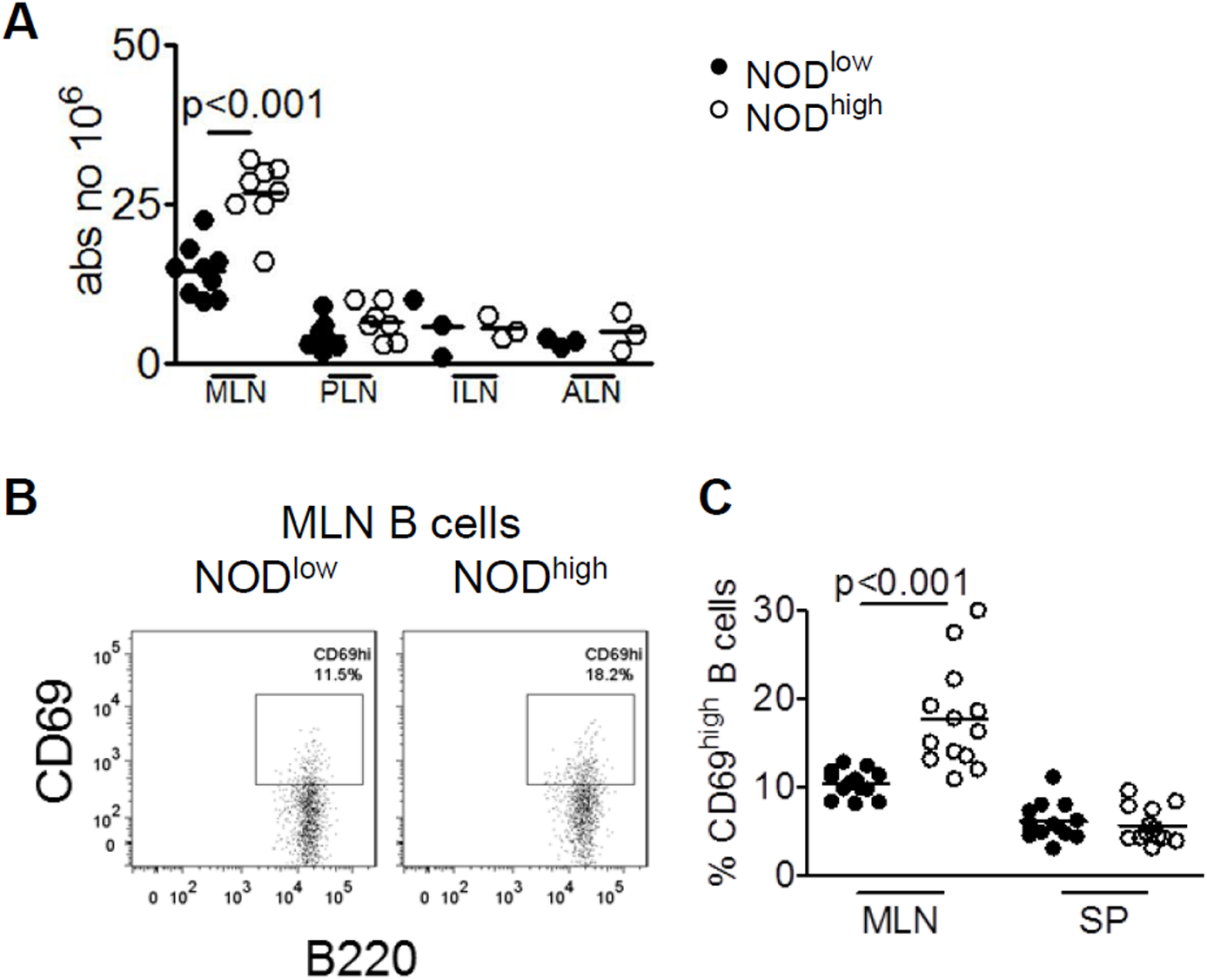
Increased B-cell activation in mesenteric lymph nodes of NOD^high^ mice. (**A**) Total cell counts in the indicated lymphoid organs of NOD^low^ (closed circles) vs. NOD^high^ (open circles) mice: MLN = mesenteric lymph nodes, PLN = pancreatic lymph nodes, ILN = inguinal lymph nodes. Analysis by 2-way ANOVA with p values given for significant colony differences. (**B**) Representative flow cytometry dot plots of CD69^high^ B cells isolated from mesenteric lymph nodes of NOD^low^ (left) and NOD^high^ (right) females at six weeks of age. (**C**) Frequencies of CD69^high^ cells in gated B220^+^ B cells from mesenteric lymph nodes (MLN) or spleens (SP) of NOD^low^ (closed circles) vs. NOD^high^ (open circles) female mice. Individual mice, means and significant p values (p < 0.05, by 2-way ANOVA) are shown.

We hypothesized that persistence of several pathobionts in the NOD^high^ colony might be associated with increased immune activation in gut-related lymphoid organs. Indeed, young NOD^high^ females had greater numbers of mesenteric lymph node (MLN) cells than NOD^low^ mice (Fig 4A). In contrast, no colony differences were observed in the cell counts of inguinal, axillary, or pancreatic lymph nodes. Interestingly, the percentage of B cells expressing the early activation marker CD69 was increased in MLN from NOD^high^ mice, but not spleen (Fig 4B and 4C). Therefore, our data suggested that B cells in the MLNs of young female NOD^high^ mice respond more actively to local stimulation, well before the onset of overt autoimmunity.

Even though the activation phenotype appeared to be restricted to MLN, it was possible that B cell immune homeostasis was perturbed more broadly in NOD^high^ mice. To address this possibility, we measured the concentration of serum B cell activating factor (BAFF), a cytokine that plays a key role in B cell activation as well as maturation and is elevated in autoimmune conditions [47, 48]. The level of BAFF was increased in NOD^high^ compared to NOD^low^ females at 6 weeks of age (S2 Fig), consistent with altered B-cell homeostasis as well as with B-cell activation.

### Exposure of NOD^low^ mice to NOD^high^ mice after weaning does not increase T1D incidence

**Fig 5:**
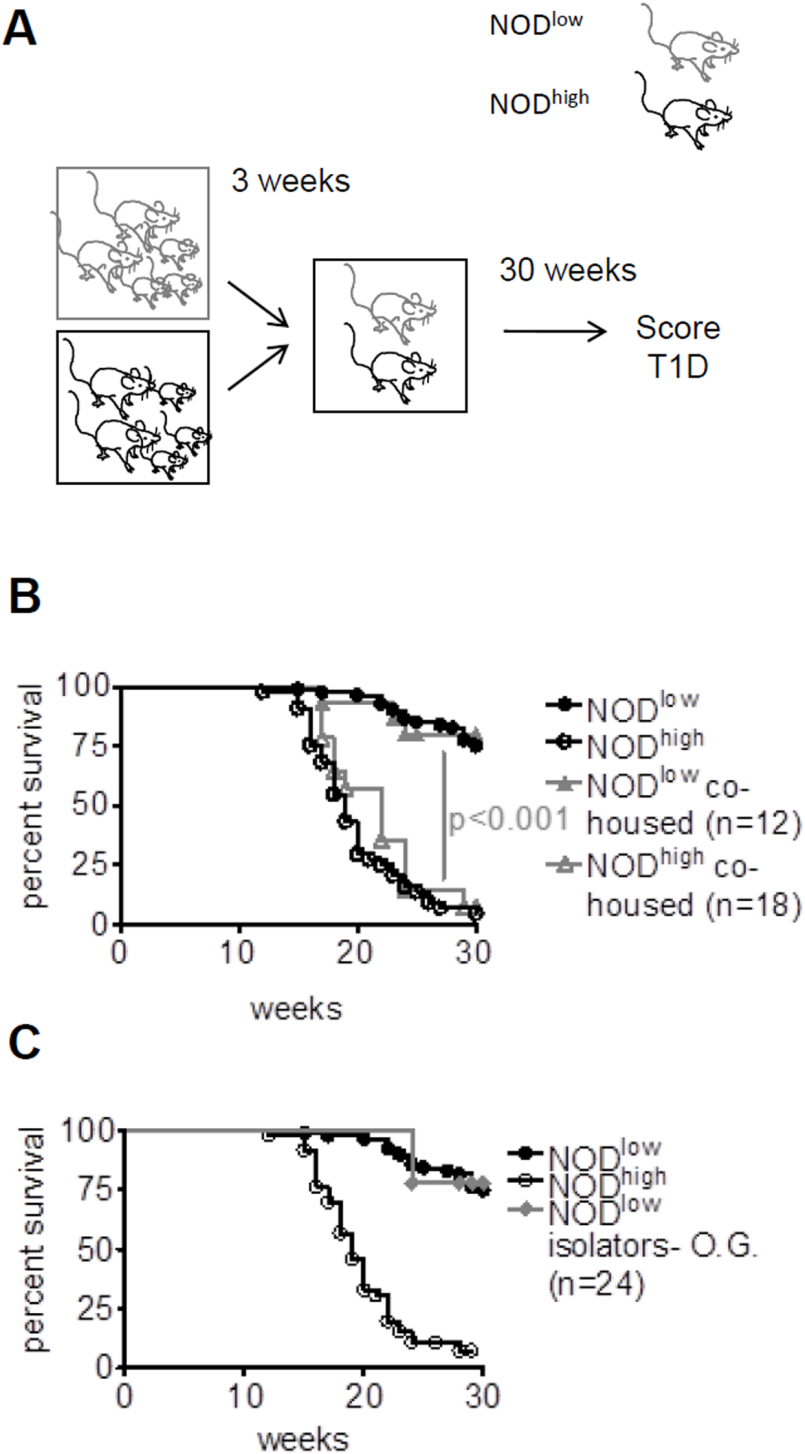
Exposure to a diabetogenic environment after the age of weaning does not modify T1D development in NOD^low^ mice. (**A**) Schematic illustration of continuous co-housing of NOD^low^ (grey outline) with NOD^high^ (black outline) mice from 3 weeks of age onwards. (**B**) Kaplan-Meier analysis of diabetes-free survival in females up to 30 weeks of age, comparing the original NOD^low^ (black circles, n = 85) and NOD^high^ (white circles, n = 45) colonies (cf. Fig 1A) with NOD^low^ (full grey triangles) and NOD^high^ females (empty grey triangles) that were co-housed from three weeks of age. Despite being in a shared environment, the animals retained the disease incidence curves of their colonies of origin. (**C**) Diabetes-free survival in females from the original colonies (symbols as in **B**) was compared with that of NOD^low^ mice orally gavaged with fecal matter collected from 12-week-old female pre-diabetic NOD^high^ mice (closed grey diamonds) at three weeks of age and maintained in isolators. Fecal gavage did not raise T1D incidence over that in the NOD^low^ parental colony (p > 0.05, log rank test), despite successful transmission of *H*. *hepaticus* (cf. S3 Fig).

To examine the transmissibility of colony differences in T1D development, 3-week-old female NOD^low^ weanlings were co-housed with sex- and age-matched NOD^high^ mice, and both groups were monitored for T1D up to 30 weeks of age (Fig 5A). Co-housed NOD^low^ and NOD^high^ mice developed T1D with the same cumulative incidence as their parental colonies (Fig 5B), indicating that exposure to the NOD^high^ environment after the age of weaning had no effect on the course of the disease, and *vice versa*.

This experiment did not rule out the possibility that the relevant environmental factor was difficult to transmit by co-housing. In order to examine another method of exposure, NOD^low^ weanlings were orally gavaged with faecal matter obtained from 12-week-old pre-diabetic NOD^high^ females and were kept in dedicated isolators until they reached 30 weeks of age. Acquisition of *H*. *hepaticus* was verified by PCR (S3 Fig). Nonetheless, these mice developed T1D with the same low rate of incidence shown by the NOD^low^ colony (Fig 5C).

These data show that, after the age of weaning, exposure to transmissible factors in the NOD^high^ environment, including some known pathobionts, does not raise T1D incidence in NOD^low^ mice. This was surprising because post-weaning exposure to various microbial or viral species has previously been shown to alter T1D incidence in NOD mice [21, 23, 24, 49].

### Offspring of co-housed NOD^low^ mice acquire increased T1D incidence, microbiota and immune phenotypes similar to NOD^high^ mice

**Fig 6:**
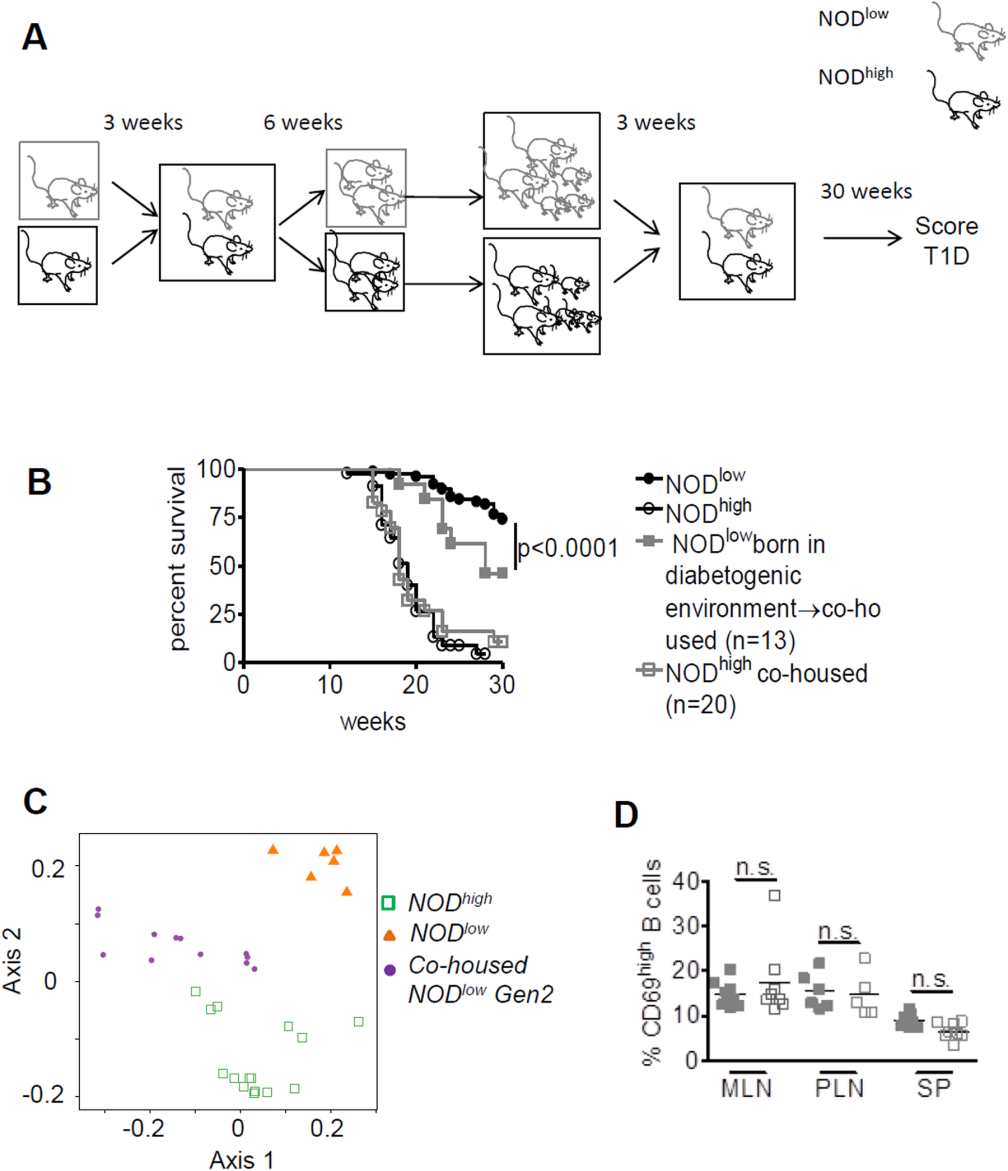
Increased T1D incidence and acquisition of NOD^high^-like B-cell phenotypes in female offspring of co-housed NOD^low^ mice. (**A**) Scheme illustrating co-housing of NOD^low^ with NOD^high^ mice from 3 weeks of age, followed by breeding and continued co-housing of offspring from their age of weaning. (**B**) Kaplan-Meier analysis, comparing diabetes-free survival in females up to 30 weeks of age between the original NOD^low^ (black circles, n = 85) and NOD^high^ (white circles, n = 45) colonies (cf. Fig 1A), and between the offspring of co-housed NOD^low^ (closed grey squares) and co-housed NOD^high^ (open grey squares) mice. Survival curves for the offspring of co-housed animals were compared to the appropriate colony of origin by log rank test. (**C**) Intestinal microbiota (by metagenomic analysis) at 5 weeks of age in the offspring of co-housed NOD^low^ mice (filled purple circles), compared with those in the original NOD^low^ (filled orange triangles) and NOD^high^ (open green squares) colonies. Principal Coordinates Analysis was performed, with the top two components displayed. Each group of mice clusters separately, with the offspring of co-housed NOD^low^ animals intermediate between the two original colonies. See also S1 Fig. (**D**) CD69 expression in B cells from mesenteric lymph nodes (MLN), pancreatic lymph nodes (PLN), and spleens (SP) obtained from 6-week-old NOD^low^ females (grey full squares) and NOD^high^ females (grey empty squares). Group means were compared by 2-way ANOVA with post-test for colony differences.

We hypothesized that, in order to affect T1D development, exposure to the diabetogenic environmental factor(s) has to occur prior to weaning. To test this hypothesis, sex- and age-matched NOD^low^ and NOD^high^ mice were co-housed in the NOD^high^ animal facility, beginning at three weeks of age. The co-housed mice were separated at 6–7 weeks for breeding. Their offspring were then co-housed again, starting at three weeks of age, and T1D development was monitored up to 30 weeks of age (Fig 6A). While the offspring of co-housed NOD^high^ mice maintained high rates of T1D incidence, the offspring of co-housed NOD^low^ mice showed significantly increased T1D incidence, compared to the parental NOD^low^ colony (Fig 6B). The cumulative disease incidence was not as high as that observed in the established NOD^high^ colony; nonetheless, the data suggested that exposure to the NOD^high^ diabetogenic environment throughout the animals’ life span, including the pre-weaning period, had a dominant effect on increasing T1D development in these mice.

In order to examine whether the offspring of co-housed NOD^low^ mice had acquired NOD^high^-like gut microbiota, intestinal 16S rRNA gene sequences from these mice were compared with animals from the original NOD^high^ and NOD^low^ colonies at 5 weeks of age (Fig 6C and S1 Fig). Fig 6C shows a Principal Coordinates Analysis, a statistical technique that represents differences in microbiota between any pair of mice as a two-dimensional map (see Materials and Methods and [38, 39]). This analysis clearly showed that the gut microbiota of the offspring of co-housed NOD^low^ mice was markedly different from the original NOD^low^ colony, approaching that of NOD^high^ mice (Fig 6C). Examination of the statistically significant species differences showed complete or partial acquisition of most of the species prevalent in the established NOD^high^ colony but not in the original NOD^low^ colony, including *Helicobacter* spp. (S1 Fig). Of the two genera present in the original NOD^low^ but not in the NOD^high^ colony, *Anaerostipes* was displaced in the offspring of co-housed NOD^low^ animals, whereas *Anaeroplasma* was retained (S1 Fig).

The MLNs in the offspring of co-housed NOD^low^ animals also seemed to approach the more activated phenotype of the NOD^high^ colony. CD69 expression in mesenteric lymph node B cells from second-generation co-housed NOD^low^ mice was similar to that detected in established NOD^high^ mice (Fig 6D).

In summary, NOD^low^ animals co-housed with NOD^high^ animals (or successfully infected with at least some of their pathobionts by other routes) from weaning age exhibited no change in their T1D incidence in the first generation. In the second generation of co-housing, however, the NOD^low^ animals partially acquired the increased T1D incidence and altered intestinal microbiota of the established NOD^high^ colony, correlating with increased MLN B-cell activation.

## Discussion and conclusions

In genetically predisposed individuals, development of T1D is regulated by environmental factors. Specifically, the progression to overt diabetes in the NOD mouse model is markedly influenced both by the commensal intestinal flora and by infection with pathogens [23–27, 49]. This study, a comparison of two colonies of NOD mice with very different rates of T1D incidence in females, revealed novel aspects of environmental regulation of autoimmunity. The NOD^high^ colony was shown to harbour a transmissible diabetes-promoting factor (or a combination of such factors), which differs from previously-described microbial influences. It acts during a critical, early window of susceptibility, prior to weaning age, and overcomes the apparent inhibition of T1D by SFB. Elevated T1D incidence in this system correlates with local activation of B cells. After weaning, however, NOD^low^ mice maintain T1D resistance through PD-L1 signalling, even when T_reg_ cells are depleted.

### A dominant diabetogenic transmissible factor in NOD^high^ mice

The natural history of the two colonies suggested an environmental influence that, over several generations of breeding, raised the cumulative T1D incidence in NOD^low^-derived mice to the high incidence (≈ 90% by 30 weeks of age) found in the established NOD^high^ colony. Both colonies had comparable levels of insulitis, and PD-L1 blockade unmasked latent autoimmunity in NOD^low^ females, suggesting that both colonies had autoimmune potential. Transmissibility of the differences in T1D incidence was examined co-housing NOD^low^ and NOD^high^ animals. Female offspring of co-housed NOD^low^ animals, exposed to NOD^high^-derived environmental factors throughout their life span, developed T1D more frequently (≈ 50% by 30 weeks of age), than their dams or the original NOD^low^ colony (≈ 20%). In contrast, co-housed offspring of NOD^high^ animals retained their high (90%) cumulative T1D incidence. Thus, the increased T1D incidence in 2^nd^-generation co-housed NOD^low^ mice was a dominant phenotype, caused by acquisition of a transmissible factor (or factors) from the NOD^high^ colony.

These findings suggested an infectious cause. Indeed, the NOD^high^ colony harbored several microbes absent from the NOD^low^ colony. This included *Helicobacter* spp. (*hepaticus* and *typhlonius*), which establish persistent, asymptomatic infection in immunocompetent mice [50], as seen here, but trigger colitis in immunodeficient models [51–54]. *Prevotella copri* was ≈ 10× more abundant in the NOD^high^ colony; this bacterium is also over-represented in patients with recent-onset human rheumatoid arthritis [55]. These species may be diabetogenic in our system. As for viruses, MNV was present only in the NOD^high^ colony, but there was no evidence that it raised T1D incidence in NOD^high^ females (S1 Appendix). Conversely, two bacterial genera were present in NOD^low^ mice but absent from the NOD^high^ colony. Of these, *Anaeroplasma* has been associated with protection of mice against a sex-dependent autoimmune demyelinating disease [56]. Thus, displacement of diabetes-protective species might also contribute to the increased T1D development in the NOD^high^ environment.

The 2^nd^-generation co-housed NOD^low^ animals did not reach the very high cumulative disease incidence of female NOD^high^ mice (50% vs. 90% at 30 weeks). This was reminiscent of the gradual rise in disease incidence over generations after the NOD^high^ colony was first established. These animals acquired only some of the NOD^high^–specific microbiota. Thus, multiple diabetes-promoting and dominant-acting changes in microbial composition may be required for the high T1D incidence of established NOD^high^ mice, some of which may require multiple generations of exposure.

### Is early exposure to the NOD^high^-derived transmissible factor critical?

Surprisingly, we were repeatedly unable to increase T1D incidence by transferring microbiota to NOD^low^ mice after the age of weaning, whether by oral gavage or co-housing. Moreover, both *Helicobacter* spp. and MNV were consistently found in the recipients’ feces shortly after exposure. Thus, at least the known pathobionts were readily transmissible, presumbably by coprophagy [57–59], and survive gastric passage to establish persistent infection.

In contrast, an increase in the frequency of T1D development was seen in the offspring of co-housed NOD^low^ mice. These animals experienced the diabetogenic environment throughout their entire life span, including during gestation and/or during suckling. Thus, exposure to the NOD^high^ environment may promote autoimmunity only during a critical window of susceptibility early in immune development. Thereafter, NOD^low^ mice seem to resist the diabetogenic influences within the NOD^high^ colony.

The need for pre-weaning exposure to raise T1D incidence in our system is novel. Many previous studies showed that microbial exposures in adulthood, well after the age of weaning, are sufficient to affect T1D development in NOD mice [23, 24, 26, 27, 49, 60–62]. Antibiotic treatment of pregnant NOD dams creates a simplified microbiome and increases T1D development in their offspring [27], but that study did not examine the effects of post-weaning exposure.

Other explanations of our data are possible. For example, the diabetogenic factor(s) might be poorly transmissible after weaning, or slow to reach pathogenic levels in the gastrointestinal tract, so that the exposure in the first generation of co-housing could be insufficient to raise T1D incidence. Alternatively, increased disease incidence may require vertical transmission of the responsible infectious agent(s) or co-transfer of host factors (such as autoantibodies) from exposed parents to offspring, or dilution of epigenetic marks carried over from the first generation. None of these possibilities contradict the need for an early-acting environmental factor. Moreover, as discussed further below, the immunophenotypic analysis was consistent with a window of susceptibility early in immune development.

### B-cell activation in MLN correlates with high T1D incidence

The pathogenesis of T1D involves complex interactions of many immune components, both in rodent models and in humans [23, 63]. Thus, we explored immune correlates of the environmental differences in T1D incidence in our system.

Classically, T1D in NOD mice and BB-DP rats has been considered to be a T cell-driven disease [64, 65]. Many previously-described microbial modulators of NOD autoimmunity act *via* T_reg_ cells or regulatory cytokines [23]. In this study, however, we found no evidence for major differences in T-cell phenotypes correlating with the observed colony difference in cumulative T1D incidence. Splenic T-cell percentages and counts were similar between the NOD^high^ and NOD^low^ colonies (S4 Fig). Moreover, T_reg_ cells were not responsible for the colony difference in T1D development, as T_reg_ cell frequencies did not differ between the colonies, and the difference in T1D incidence persisted when T_reg_ cells were depleted by cyclophosphamide [33]. In contrast, we observed a clear colony difference in mesenteric lymph nodes, especially in B cells. MLN cell counts were increased in NOD^high^ mice, with increased CD69 expression in B cells, consistent with greater activation by intestinal pathobionts. Interestingly, this phenotype also correlated with increased T1D incidence in the offspring of cohoused NOD^low^ mice. The difference was clearly established by 6 weeks of age, consistent with an early-acting environmental factor being responsible.

A mechanistic link between increased MLN B-cell activation and increased rates of T1D development is plausible, because B cells are key mediators and regulators of autoimmunity in general [66], and this extends to NOD mice. Genetic ablation of B cells prevents T1D [67]; antibody-mediated depletion of B cells ameliorates the disease in mice [68, 69] and delays T1D progression in human prediabetic children [70]. Autoreactive B cells may present islet antigens to self-reactive T cells, whereas mechanisms depending on autoantibody secretion are thought to be less important [71–73]. NOD mice have impaired peripheral B cell tolerance at the transitional check point [74], which involves BAFF (also known as BLyS), a B-cell stimulating cytokine elevated in autoimmune conditions [75]. Moreover, genetic and phenotypic studies implicate TACI, one of the BAFF receptors, in type 1 diabetes development [76]. Given the elevated BAFF levels in NOD^high^ sera, such mechanisms may raise T1D incidence in the NOD^high^ environment. Interestingly, B cells have recently been implicated in rotavirus acceleration of T1D in NOD mice [60], though in contrast to the present study, rotavirus is diabetogenic in adult animals.

We considered the further possibility that immune regulation by B cells might be impaired in NOD^high^ mice. In the NOD strain, the characterisation of regulatory B cells (B_reg_ cells) has been difficult due to a paucity of informative markers [77], but IL-10-producing B cells enriched from long-term normoglycaemic NOD mice exhibit immunoregulatory properties [78]. To address a possible contribution of this mechanism in our system, we measured IL-10 secretion by LPS-stimulated B cells *in vitro*, but found no functional impairment of B_reg_ cells from NOD^high^ mice on a per-cell basis (S5 Fig).

Signalling by PD-1/PD-L1, an immunoregulatory ligand/receptor pair [79], could also be involved. Both the ligand and the receptor are expressed on T cells and, inducibly, on B cells [79]. We found by antibody blocking that PD-L1 signalling maintains low rates of T1D development in NOD^low^ mice. Similarly, previous work had shown that PD-L1 blockade unmasks T1D in pre-diabetic NOD^high^ mice and reverses suppression of T1D by *Salmonella typhimurium* [32]. PD-L1 mediates local immune regulation in pancreatic lymph nodes at a young age, and systemic regulation in older prediabetic NOD mice [46]. Prevention of T1D in NOD mice by PD-L1 signalling does not require B cells [46], but this does not argue against a role for B-cell activation in our NOD^high^ colony.

In conclusion, the activation of B cells in NOD^high^ MLNs, possibly driven by interaction with the diabetogenic factor(s) in this colony, correlates closely with increased T1D development due to the NOD^high^-derived diabetogenic factor(s). This phenotype emerges at a young age, consistent with the early window of susceptibility, and may be related to increased BAFF levels in NOD^high^ sera, PD-1/PD-L1 signalling, or both. Further work is required to address these possibilities.

### A hierarchy of microbial influences on progression to T1D?

This study revealed unexpected complexity in the environmental regulation of T1D. First, our observations do not fit well with the Hygiene Hypothesis (cf. Introduction): NOD^high^ mice exhibit high T1D incidence despite harbouring a somewhat more complex microbiome (including several pathobionts) than NOD^low^ mice. Rather, the NOD^high^ microbiome includes a novel, dominant-acting transmissible factor(s), raising T1D incidence. This adds to the many known microbial and viral influences that promote or inhibit autoimmunity (cf. Introduction).

Surprisingly, NOD^high^ mice exhibited their high T1D incidence despite harbouring SFB. Previous work had correlated SFB colonisation with low rates of T1D development [22], suggesting a dominant suppressive effect of SFB. The suppressive effect in T1D contrasts with the ability of SFB to enhance Th17-driven autoimmunity in other models [80], and has been linked to altered Th1/Th17 balance in the intestine. In our studies, SFB suppression of T1D in NOD^low^ mice seems to be overcome by the diabetogenic NOD^high^-derived environmental factor(s).

Taken together with prior literature, our findings suggest a hierarchy of diabetes-promoting and suppressive influences, each acting via distinct immune mechanisms. In order from low to high microbial complexity and dominance, these are, at a minimum: sex-independent high incidence in germ-free mice [24]; sex-dependent metabolomic influences of microbiota, suppressing T1D in male mice [24]; Th1-to-Th17 cytokine deviation by SFB, suppressing T1D in females [22]; the NOD^high^-derived environmental factor(s), which stimulate(s) mesenteric LN B cells and raises female T1D incidence (this study); and the T1D-suppressive influences of IL-10 production stimulated by parasites, or of PD-L1 signalling stimulated by *Salmonella* in NOD^high^ mice [20, 21]. This mechanistic diversity is further supported by the different developmental windows of susceptibility to diabetes-promoting and -suppressive influences, discussed above.

### Translational implications

Environmental influences on autoimmunity differ between humans and mice, but parallels may exist. As in mice, human T1D is associated with altered intestinal microbiota (cf. Introduction), and infections influence T1D risk (reviewed in [23]). As in our present study of NOD mice, dominance hierarchies may also characterize environmental influences on human autoimmunity, acting via different immune mechanisms, with some only being effective during a particular developmental window of susceptibility. This may complicate health prognoses based on the composition of intestinal microbiota: if the presence of some microbial species may exert dominant effects, additive models of these influences are insufficient. The possible role of local B-cell activation in the environmental regulation of T1D development merits further study, both in mice and humans. Importantly, our data show that some diabetogenic environmental factors may be acquired asymptomatically by older recipients, without altering their own disease outcomes, yet may increase the potential for autoimmunity if acquired early enough in life and/or transmitted vertically to future generations. If the same applies to humans, this would complicate the identification of viral or microbial species that regulate autoimmunity. It also would raise the possibility of unforeseen autoimmune complications in the children of patients who have received fecal transplants, an emerging therapy for intractable infections, such as antibiotic-resistant *Clostridium difficile*, and for other inflammatory diseases [81].

### Concluding remarks

The ‘Hygiene Hypothesis’ no longer seems sufficient to account for the complex hierarchy of immune mechanisms by which multiple microbial and viral factors influence T1D development. Developmental timing and the role of B-cell activation must be considered when considering microbial regulation of T1D. Potentially, human parents could transmit an increased risk of autoimmunity to their offspring upon vertical transmission of infectious agents acquired asymptomatically.

## Supporting information

S1 Appendix Further experiments on the possibility of a diabetogenic effect of MNV. Contains brief introduction, methods, results and discussion of experiments showing that MNV infection of NOD^low^ mice fails to raise their low T1D incidence.
(PDF)

S1 Fig Abundance of selected intestinal bacteria in individual mice assessed by high-throughput sequencing. Counts for specific bacterial 16S rRNA gene sequences were normalized by sequencing depth, showing significant differences in any pairwise comparisons of intestinal bacterial composition between 5-week-old female NOD^high^ mice (green open squares), NOD^low^ mice (orange closed triangles), and the offspring of NOD^low^ mice co-housed with NOD^high^ mice (LowBornHigh, purple closed circles). Data for individual mice and means are shown for each group.
(PDF)

S2 Fig Increased BAFF levels in the serum of NOD^high^ mice. BAFF concentrations in the sera of NOD^low^ (closed circles) and NOD^high^ (open circles) assessed by ELISA. Sera from individual mice are shown, and means were compared by Student’s t test.
(PDF)

S3 Fig Three-week-old NOD^low^ female weanlings acquire *H*. *hepaticus* following oral gavage. Gel electrophoresis showing PCR amplification of *H*. *hepaticus* genomic DNA isolated from feces of NOD^low^ mice orally gavaged at weaning (3 weeks old) with a fecal suspension obtained from 12-week-old pre-diabetic NOD^high^ females. Lane 1: DNA marker; lane 2: negative control; lanes 3 and 4: representative NOD^low^ recipients.
(PDF)

S4 Fig Similar T-cell frequencies and absolute counts in splenocytes from NOD^low^ and NOD^high^ mice. Splenocytes were obtained from six-week-old female mice from both colonies (n = 8 each), counted, stained for CD3, and analysed by flow cytometry. Percentages (left) and absolute counts (right) of CD3+ T cells are shown for individual NOD^low^ (black circles) and NOD^high^ (white circles) animals; horizontal bars represent means.
(PDF)

S5 Fig Similar IL-10 secretion by LPS-stimulated splenic B cells from NOD^low^ and NOD^high^ mice. Splenocytes were obtained from six-week-old female mice from both colonies and used for immunomagnetic (MACS) enrichment of B cells to high purity (> 97% CD19^+^B220^+^; (**A**)). IL-10 release following stimulation with LPS (10 μg/ml) was quantified by ELISA (**B**). Data are shown for individual NOD^low^ (black circles) and NOD^high^ (white circles) animals; horizontal bars represent means.
(PDF)
