# Supplementary material for: Regulation of type 1 diabetes development and B-cell activation in nonobese diabetic mice by early life exposure to a diabetogenic environment
Source: PLoS One. 2017 Aug 3;12(8):e0181964. doi: 10.1371/journal.pone.0181964 (PMC5542673; doi:10.1371/journal.pone.0181964)
Supplement: S1 Appendix — Contains brief introduction, methods, results and discussion of experiments showing that MNV infection of NODlow mice fails to raise their low T1D incidence. (PDF) [file pone.0181964.s001.pdf]

S1 Appendix

Further experiments on a potential diabetogenic effect of MNV

Even though MNV was not the cause of the original rise in T1D penetrance in the NOD^{high} colony (see main text, Results), we nonetheless investigated whether *de novo* introduction of MNV might be diabetogenic in NOD^{low} animals. In various wild-type and immunodeficient mouse strains, different strains of MNV can cause mild gut inflammation [1], spread systemically with or without clinical manifestations [1, 2], and exacerbate inflammation provoked by enteric bacteria [3-5]. Other viruses also have been implicated in triggering T1D (cf. main text, Introduction).

We confirmed the presence of MNV in the NOD^{high} colony, and its absence in the NOD^{low} colony, by PCR (panel A of the Figure). We further investigated whether infection of NOD^{low} mice with a persistent strain of MNV (MNV-3) by oral gavage at the time of weaning was diabetogenic, as had been shown for other viruses (cf. Introduction). However, the T1D penetrance of MNV-3-infected NOD^{low} weanlings remained indistinguishable from that of uninfected NOD^{low} controls when followed up to 30 weeks of age (panel B). The infected mice did acquire productive MNV infection, as shown by RT-PCR (panel C).

These observations do not support the idea that oral MNV-3 infection of NOD^{low} mice at the age of weaning is diabetogenic. Given that other diabetogenic viruses do act after the age of weaning and that the natural history of the NOD^{high} colony ruled out MNV as the explanation of its original slow rise in T1D incidence, we did not test whether pre-weaning exposure to MNV raised T1D incidence; this remains formally possible but seems unlikely.

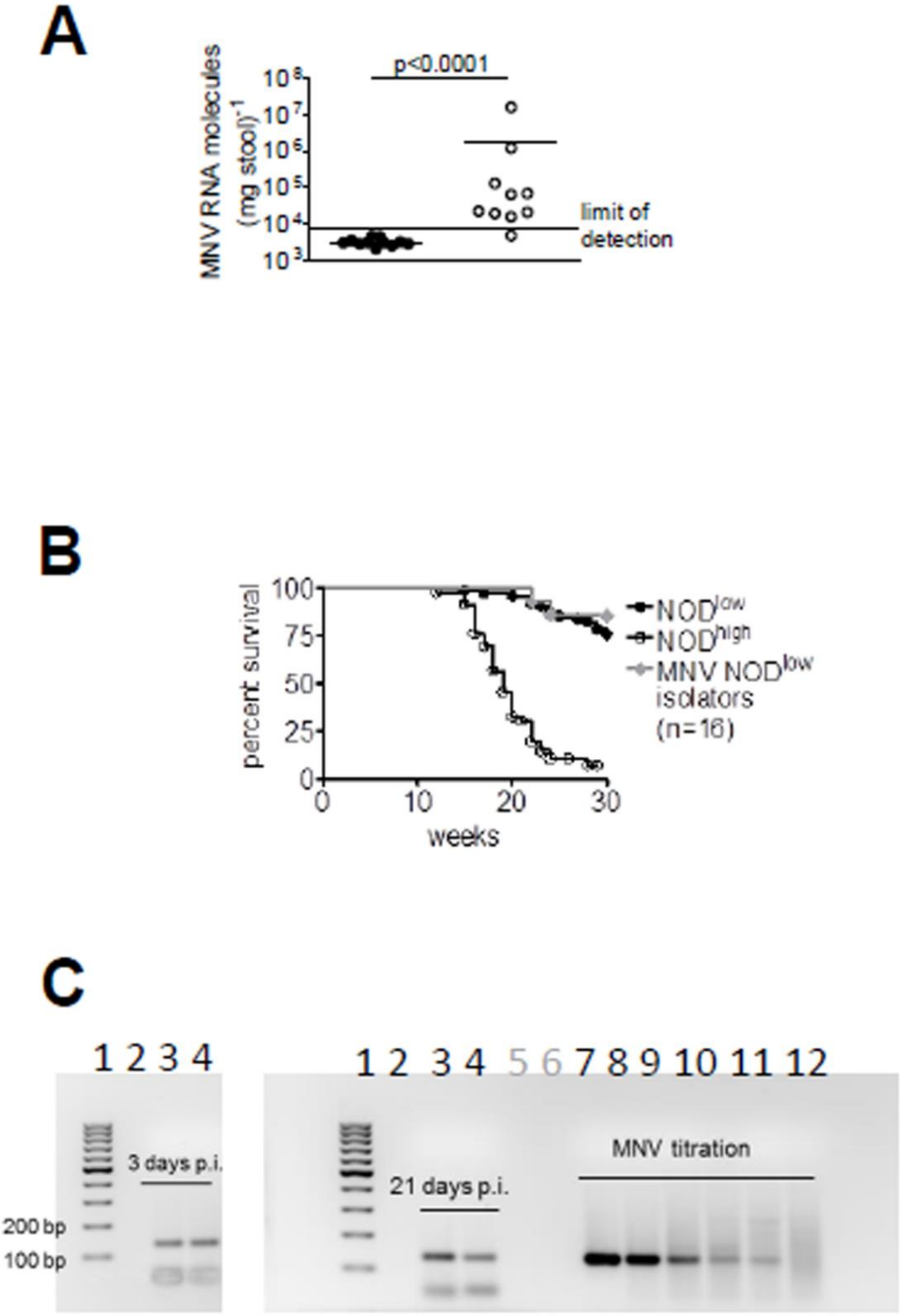

Figure. No diabetogenic effect of MNV strain 3 infection in NOD^{low} weanlings.

(**A**) Quantification by RT-qPCR of MNV3 (genome copies/mg) in the feces of NOD^{low} (black circles) and NOD^{high} (white circles) mice. Significant ($p < 0.05$) differences are indicated.

(**B**) Diabetes-free survival in females from the original colonies (symbols as in Fig 1A of the main text) was compared with that of NOD^{low} mice orally infected with MNV3 (closed grey diamonds) at three weeks of age and maintained in isolators. Infection did not raise T1D penetrance over that in the NOD^{low} parental colony ($p > 0.05$, log rank test)

(**C**) MNV RNA amplified by RT-PCR from feces of NOD^{low} weanlings orally gavaged with 10×10^4 p.f.u. of MNV-3. Gel on the left: lane 1, DNA marker; lane 2: negative control; lanes 3-4: representative NOD^{low} samples at day three post-infection. Gel on the right: lane 1, DNA marker; lane 2: negative control; lanes 3-4: representative NOD^{low} samples at day 21 post-infection; lanes 7-12: ten-fold dilutions from starting stock of 10^7 copies MNV-3.

Methods

PCR detection of viral RNA

As described previously [6], stool pellets collected from infected animals were placed on ice and dispersed into PBS to reach a final concentration of 50 mg/ml. Re-suspended feces were then centrifuged at maximum speed (15000 g) for 5 min and 100 µl supernatant was subjected to a second centrifugation step to remove any traces of fecal debris. Viral RNA was extracted from 100 µl supernatant using GenElute Mammalian Total RNA Miniprep kit (Sigma-Aldrich). The number of MNV-3 RNA molecules was quantified by PCR. Briefly, reverse transcription of MNV cDNA was performed using M-MLV reverse transcriptase (Promega) and a primer complementary to genomic positions 5345–5380. MNV cDNA was then quantified by quantitative PCR with primers spanning residues 5028–5047 (sense) and

5177–5138 (antisense), and a TaqMan FAM-TAMRA-labelled probe complementary to residues 5077–5062. Quantitative PCR determinations were carried out with Precision 2× qPCR MasterMix (Primerdesign) in a ViiA7 Real-Time PCR system apparatus (Applied Biosystems). A standard curve for MNV RNA with a known number of molecules was carried out in parallel. Alternatively, the 150 bp PCR products were resolved on a 2% agarose gel.

MNV infection

MNV-3 was administered to three-week-old NOD^{low} females as one dose of 1 x 10^4 p.f.u. in 200 µl RPMI medium, given to each recipient by oral gavage using a 24G round tip gavage needle. The solution was filtered using a 70 µm nylon cell strainer prior dosing. Mice were maintained in an isolator dedicated to this study till 30 weeks of age and scored for diabetes as described in the main text. Fresh fecal samples from individual recipients was collected at day 3 and 14 post infection and analysed for MNV positivity by PCR and visualized on a 2% agarose gel.

References

1. Mumphrey SM, Changotra H, Moore TN, Heimann-Nichols ER, Wobus CE, Reilly MJ, et al. Murine norovirus 1 infection is associated with histopathological changes in immunocompetent hosts, but clinical disease is prevented by STAT1-dependent interferon responses. Journal of virology. 2007;81(7):3251-63. PubMed PMID: 17229692.

2. Ward JM, Wobus CE, Thackray LB, Erexson CR, Faucette LJ, Belliot G, et al. Pathology of immunodeficient mice with naturally occurring murine norovirus infection. Toxicologic pathology. 2006;34(6):708-15. PubMed PMID: 17074739.

3. Lencioni KC, Seamons A, Treuting PM, Maggio-Price L, Brabb T. Murine norovirus: an intercurrent variable in a mouse model of bacteria-induced inflammatory bowel disease. Comparative medicine. 2008;58(6):522-33. PubMed PMID: 19149409.

4. Kim YG, Park JH, Reimer T, Baker DP, Kawai T, Kumar H, et al. Viral infection augments Nod1/2 signaling to potentiate lethality associated with secondary bacterial infections. Cell host & microbe. 2011;9(6):496-507. PubMed PMID: 21669398.

5. Basic M, Keubler LM, Buettner M, Achard M, Breves G, Schroder B, et al. Norovirus triggered microbiota-driven mucosal inflammation in interleukin 10-deficient mice. Inflammatory bowel diseases. 2014;20(3):431-43. PubMed PMID: 24487272.

6. Arias A, Bailey D, Chaudhry Y, Goodfellow I. Development of a reverse-genetics system for murine norovirus 3: long-term persistence occurs in the caecum and colon. The Journal of general virology. 2012;93(Pt 7):1432-41. PubMed PMID: 22495235.
