# Supplementary figures and images for: Regulation of type 1 diabetes development and B-cell activation in nonobese diabetic mice by early life exposure to a diabetogenic environment
Source: PLoS One. 2017 Aug 3;12(8):e0181964. doi: 10.1371/journal.pone.0181964 (PMC5542673; doi:10.1371/journal.pone.0181964)

S1 Fig

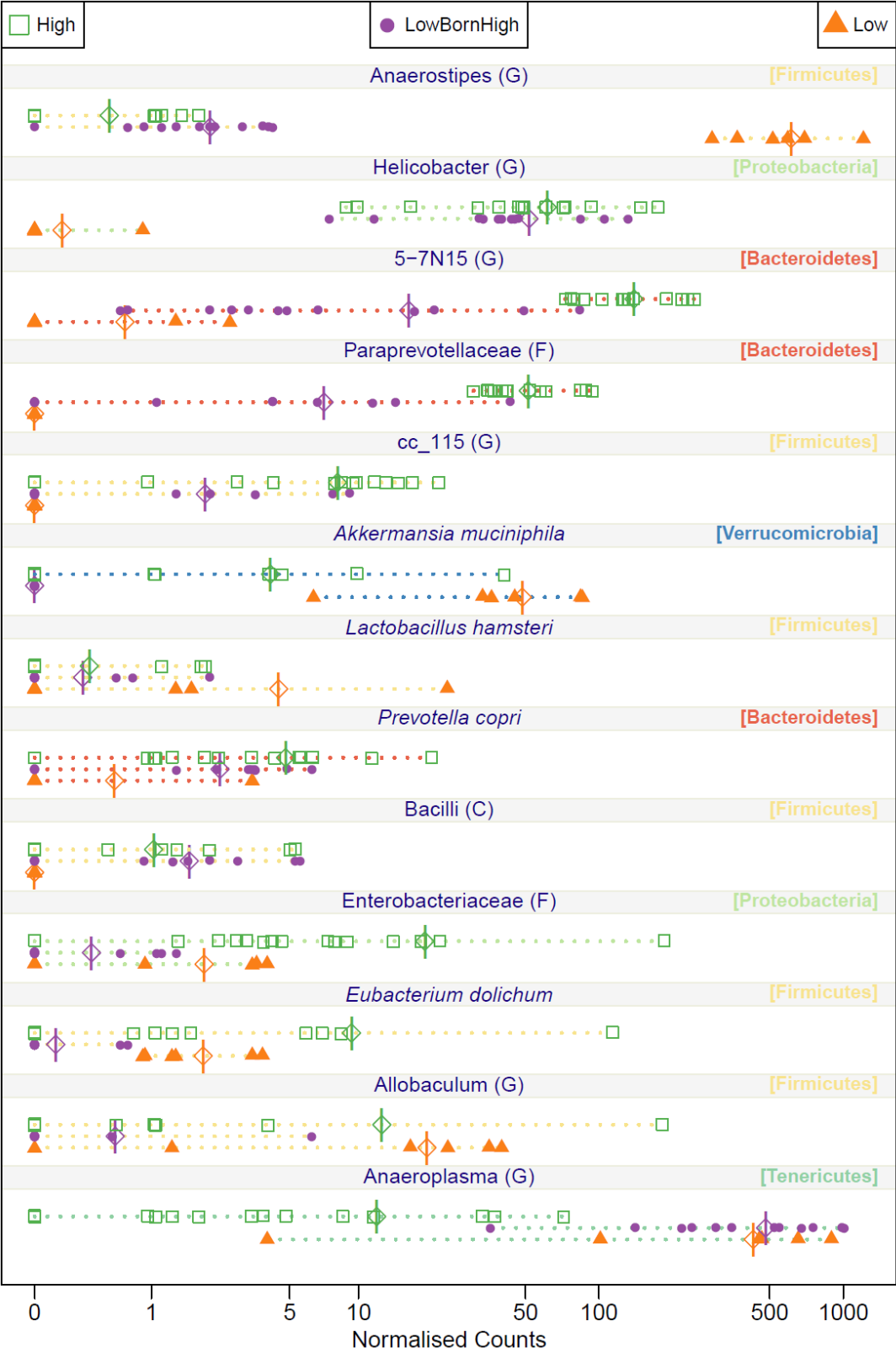

Supplement: S1 Fig — Counts for specific bacterial 16S rRNA gene sequences were normalized by sequencing depth, showing significant differences in any pairwise comparisons of intestinal bacterial composition between 5-week-old female NODhigh mice (green open squares), NODlow mice (orange closed triangles), and the offspring of NODlow mice co-housed with NODhigh mice (LowBornHigh, purple closed circles). Data for individual mice and means are shown for each group. (PDF) [file pone.0181964.s002.pdf]

S2 Fig

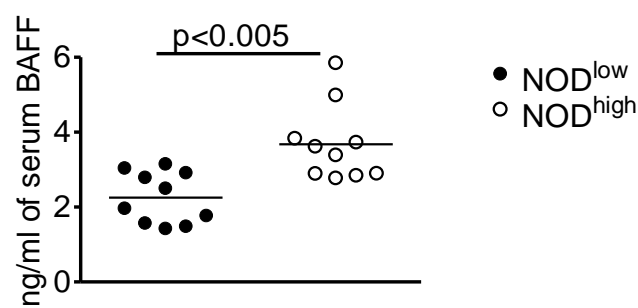

Supplement: S2 Fig — BAFF concentrations in the sera of NODlow (closed circles) and NODhigh (open circles) assessed by ELISA. Sera from individual mice are shown, and means were compared by Student’s t test. (PDF) [file pone.0181964.s003.pdf]

**S3 Fig**

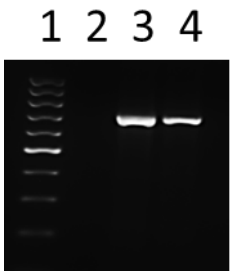

Supplement: S3 Fig — Gel electrophoresis showing PCR amplification of H. hepaticus genomic DNA isolated from feces of NODlow mice orally gavaged at weaning (3 weeks old) with a fecal suspension obtained from 12-week-old pre-diabetic NODhigh females. Lane 1: DNA marker; lane 2: negative control; lanes 3 and 4: representative NODlow recipients. (PDF) [file pone.0181964.s004.pdf]

S4 Fig

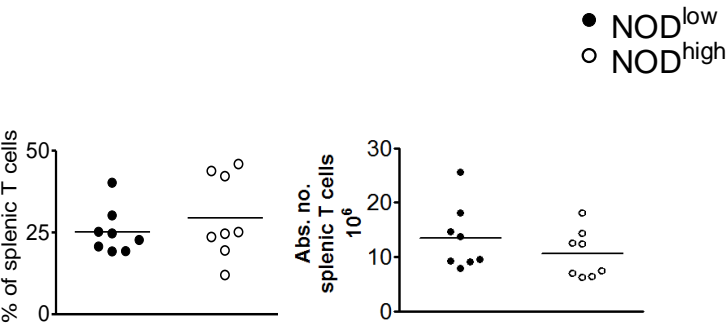

Supplement: S4 Fig — Splenocytes were obtained from six-week-old female mice from both colonies (n = 8 each), counted, stained for CD3, and analysed by flow cytometry. Percentages (left) and absolute counts (right) of CD3+ T cells are shown for individual NODlow (black circles) and NODhigh (white circles) animals; horizontal bars represent means. (PDF) [file pone.0181964.s005.pdf]

S5 Fig

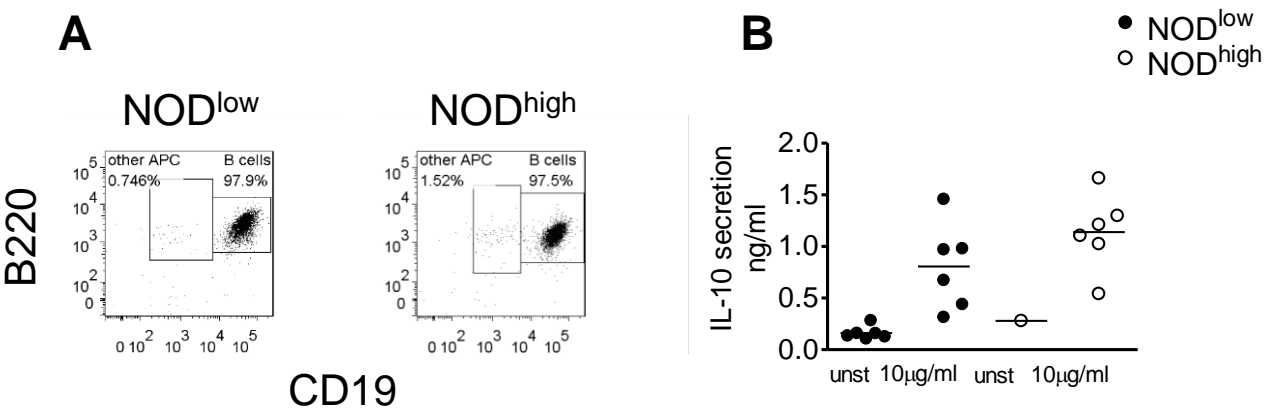

Supplement: S5 Fig — Splenocytes were obtained from six-week-old female mice from both colonies and used for immunomagnetic (MACS) enrichment of B cells to high purity (> 97% CD19+B220+; (A)). IL-10 release following stimulation with LPS (10 μg/ml) was quantified by ELISA (B). Data are shown for individual NODlow (black circles) and NODhigh (white circles) animals; horizontal bars represent means. (PDF) [file pone.0181964.s006.pdf]
